# Supplementary material for: OptoLoop – an optogenetic tool to probe the functional role of genome organization
Source: J Cell Sci. 2026 Feb 20;139(12):jcs264574. doi: 10.1242/jcs.264574 (PMC12952762; doi:10.1242/jcs.264574)
Supplement: Supplementary information [file joces-139-264574-s1.pdf]

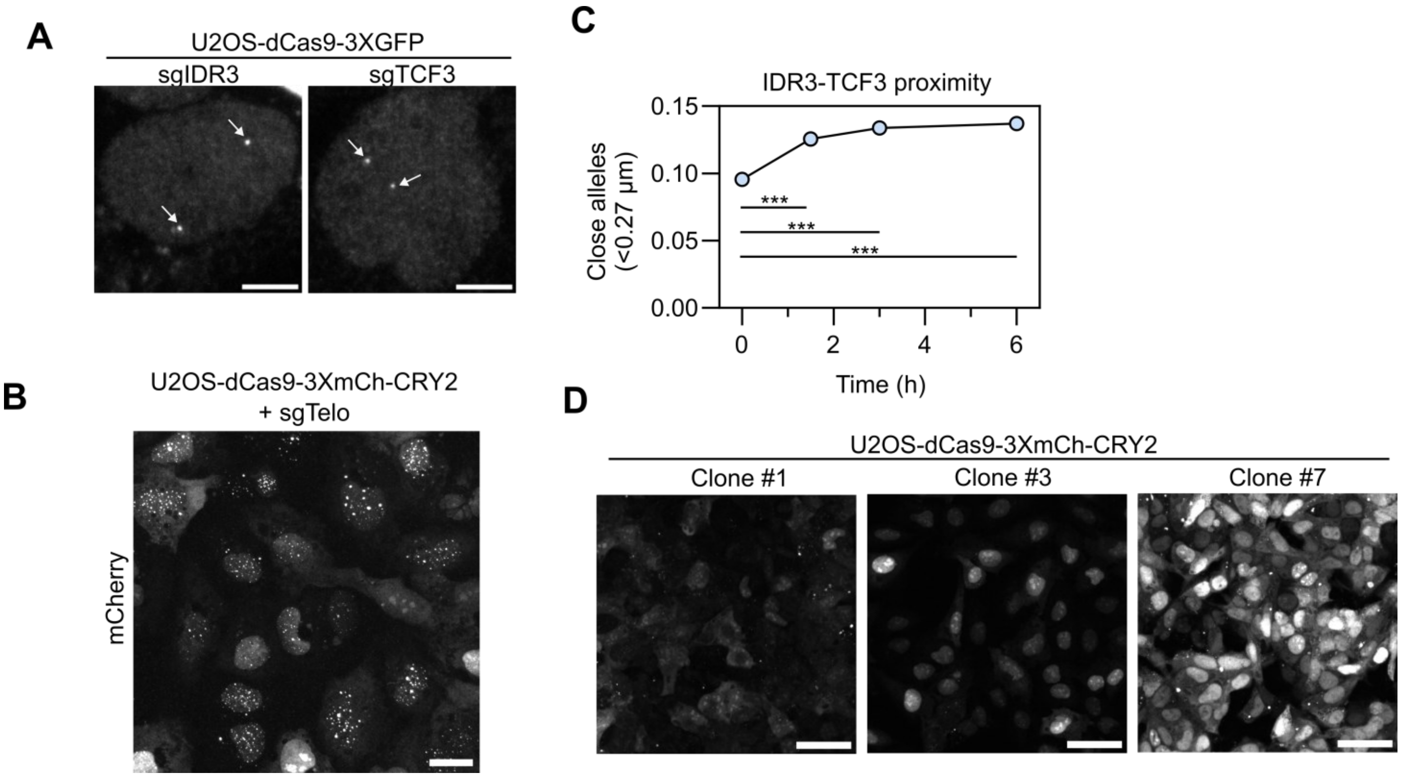

Fig. S1. Setting up OptoLoop in U2OS cells. A) Images of U2OS cells stably expressing dCas9-3XGFP and transfected with sgIDR3 and sgTCF3. White arrows indicate IDR3 and TCF3 labelled loci. Scale bar: 5 µm. **B)** Images of U2OS stably expressing dCas9-3XmCh-CRY2 (clone 3) and transfected with sgTelo. Scale bar: 20 µm. **C)** Fraction of alleles with IDR3-TCF3 distance < 0.27 µm measured from DNA-FISH images of U2OS cells stably expressing dCas9-3XmCh-CRY2 (clone 1), transfected with sgIDR3 and sgTCF3 and illuminated for variable times (1 s pulses every 10 s). Data from one experiment with typically 12000-15000 alleles analyzed per sample. **D)** Images of U2OS clones stably expressing different levels of dCas9-3XmCh-CRY2. All images are shown with the same intensity scale for comparison. Scale bar: 50 µm. Asterisks indicate significantly different comparisons (* for p<0.05, ** for p<0.01, *** for p<0.001).

Journal of Cell Science • Supplementary information

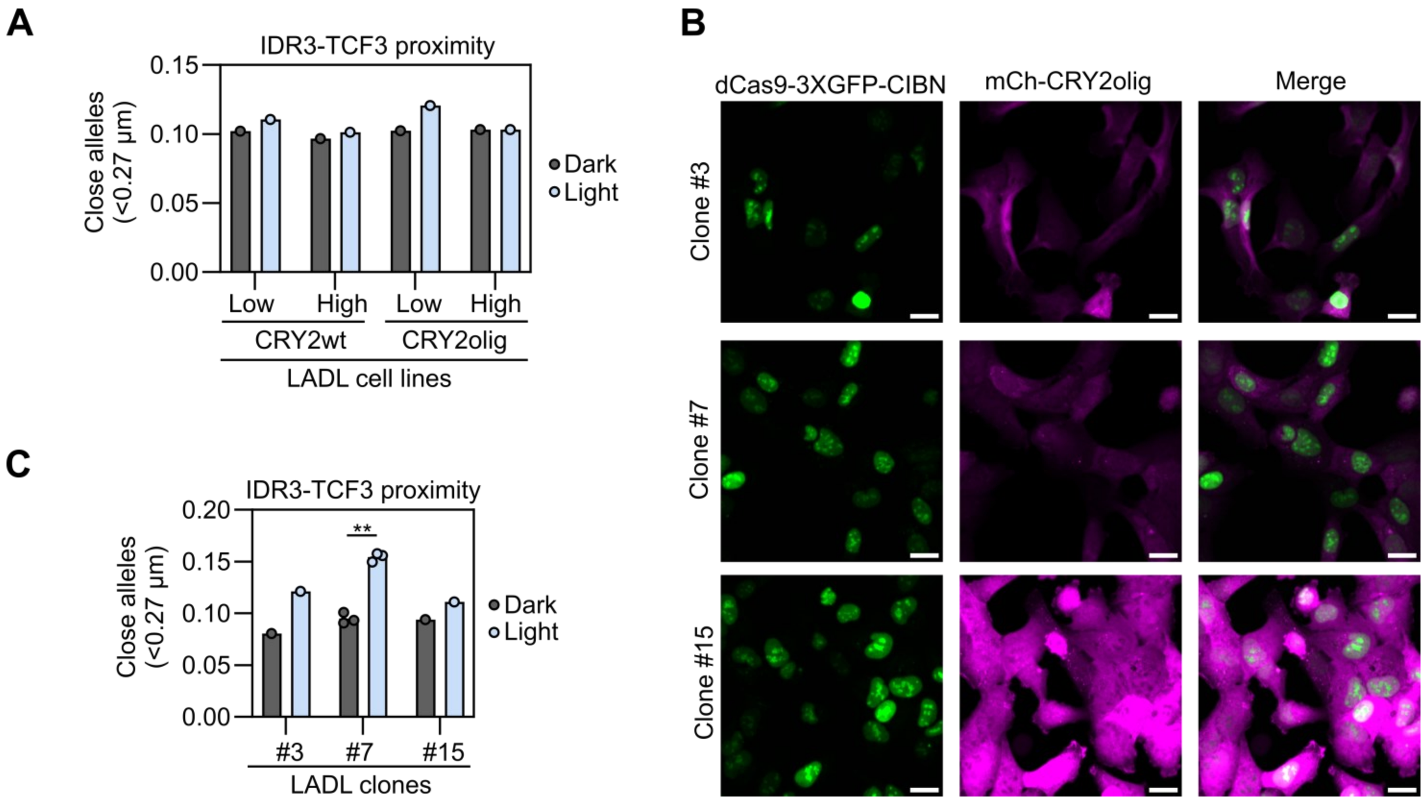

Fig. S2. Setting up LADL in U2OS cells. A) Fraction of alleles with IDR3-TCF3 distance < 0.27 µm measured from DNA-FISH images of polyclonal U2OS cell lines stably expressing dCas9-3XGFP-CRY2 and low/high expression levels of mCherry fused to CRY2wt or CRY2olig, transfected with sgIDR3 and sgTCF3, and kept under dark or illuminated with blue light for 3 h (1 s pulses every 10 s). Data from one experiment with typically 3000-5000 alleles analyzed per sample. **B)** Images of U2OS clones stably expressing different levels of dCas9-3XGFP-CIBN and mCherry-CRY2olig. All images are shown with the same intensity scale for comparison. Scale bar: 20 µm. **C)** Fraction of alleles with IDR3-TCF3 distance < 0.27 µm measured from DNA-FISH images of U2OS clones stably expressing dCas9-3XGFP-CIBN and variable levels of mCherry-CRY2olig, transfected with sgIDR3 and sgTCF3, and kept under dark or illuminated with blue light for 3 h (1 s pulses every 10 s). Each dot represents the fraction of typically 3500-7000 alleles analyzed per experiment. Bars represent the means of independent experiments. Asterisks indicate significantly different comparisons (* for p<0.05, ** for p<0.01, *** for p<0.001).

Journal of Cell Science • Supplementary information

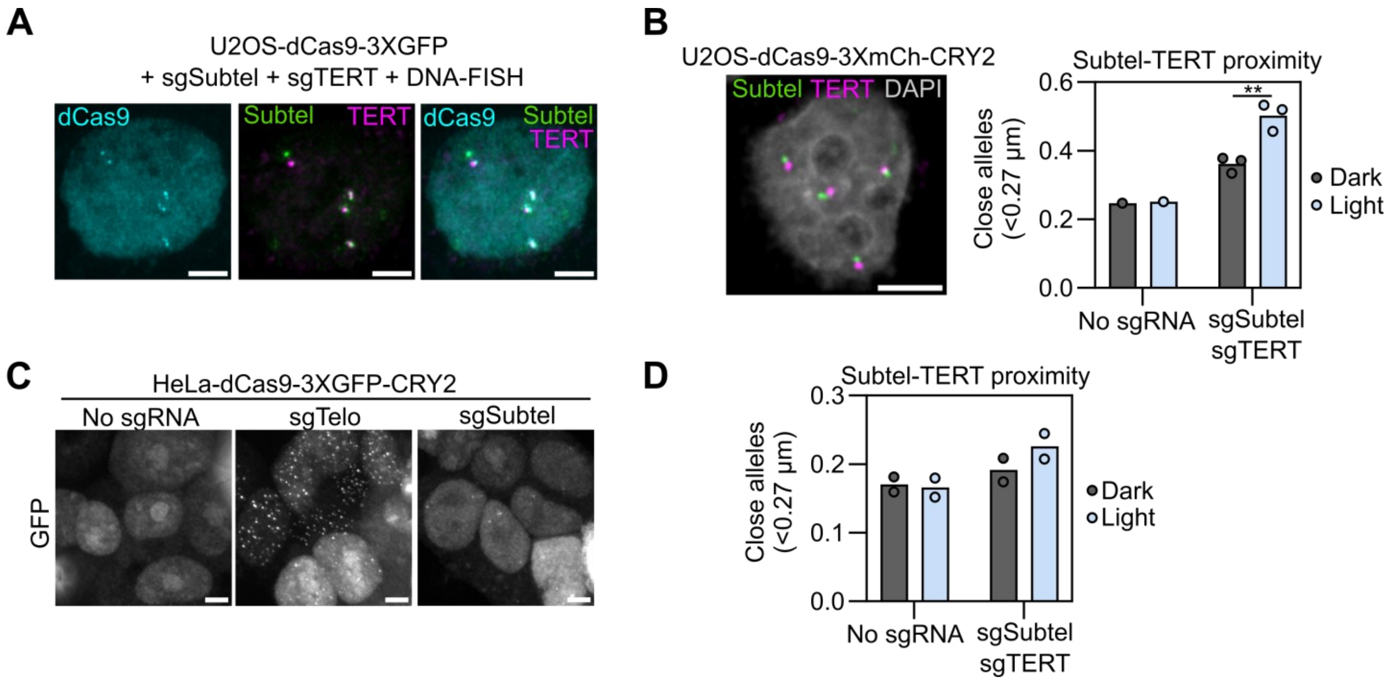

Fig. S3. Manipulation of Subtel-TERT proximity in U2OS and HeLa cells. A) Sequential images of a U2OS cell stably expressing dCas9-3XGFP and transfected with sgSubtel and sgTERT, first imaged for GFP, and then imaged after DNA FISH with Subtel and TERT BAC probes. Scale bar: 5 µm. **B)** Left panel: Representative image of DNA FISH with Subtel and TERT BAC probes in U2OS. Scale bar: 5 µm. Right panel: Fraction of alleles with Subtel-TERT distance <0.27 µm measured from DNA-FISH images of U2OS cells stably expressing dCas9-3XmCherry-CRY2 (clone 3), transfected with none or sgSubtel and sgTERT sgRNAs, and kept under dark or illuminated with blue light for 3 h (1 s pulses every 10 s). Each dot represents the fraction of typically 5000-10000 alleles analyzed per experiment. Bars represent the means of independent experiments. Asterisks indicate significantly different comparisons (* for p<0.05, ** for p<0.01, *** for p<0.001). **C)** Images of HeLa cells stably expressing dCas9-3XGFP-CRY2 and transfected with the indicated sgRNAs. Scale bar: 5 µm. **D)** Fraction of alleles with Subtel-TERT distance <0.27 µm measured from DNA-FISH images of HeLa cells stably expressing dCas9-3XGFP-CRY2, transfected with none or sgSubtel and sgTERT sgRNAs, and kept under dark or illuminated with blue light for 3 h (1 s pulses every 10 s). Each dot represents the fraction of typically 6000-20000 alleles analyzed per experiment. Bars represent the means of two experiments.

Journal of Cell Science • Supplementary information

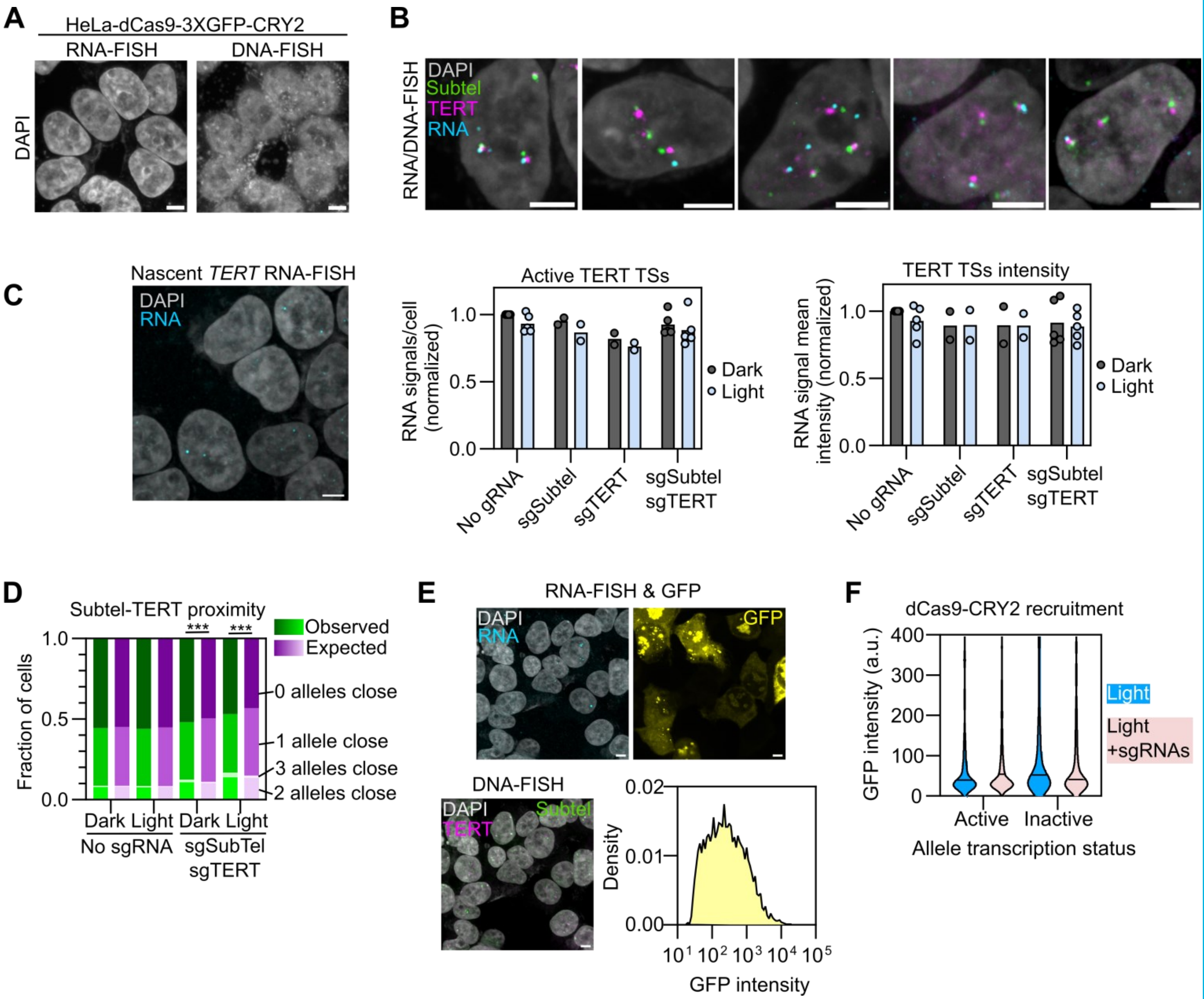

Fig. S4. Analysis of DNA looping and RNA transcription. A) Images of HeLa cells after RNA-FISH and after sequential DNA-FISH. DAPI staining is shown. Scale bar: 5 µm. **B)** Examples of RNA/DNA-FISH images of HeLa cells with TERT pre-mRNA probes and Subtel and TERT BAC probes. Scale bar: 5 µm.**C)** Left panel: Image of nascent RNA-FISH with probes against TERT intron in HeLa cells. Scale bar: 5 µm. Middle and right panels: Number of active TERT transcription sites per cell and their mean intensity, respectively, measured from RNA-FISH images from HeLa cells stably expressing dCas9-3XGFP-CRY2, transfected with the indicated combinations of sgSubtel and sgTERT, and illuminated with blue light for

Journal of Cell Science • Supplementary information

4 h (1 s pulses every 10 s). Values are represented as relative to control (no sgRNAs, no light). Each dot represents the mean of typically 3000-12000 cells analyzed per experiment. Bars represent the means of independent experiments. Differences were not statistically significant. **D)** Bars with green shades: Observed fraction of cells with none, one, two, or all three alleles with Subtel-*TERT* distance < 0.27 µm obtained from a representative DNA-FISH experiment shown in Fig. 4C with typically 6000-7000 cells analyzed per sample. Bars with magenta shades: Expected fraction of cells with none, one, two, or all three alleles with Subtel-*TERT* distance < 0.27 µm assuming that alleles from a same cell are independent of each other (Eqn. 3). Asterisks indicate significantly different comparisons (* for $p<0.05$, ** for $p<0.01$, *** for $p<0.001$). **E)** Images of sequential RNA/DNA-FISH of HeLa cells stably expressing dCas9-3XGFP-CRY2 with *TERT* pre-mRNA probes and Subtel and TERT BAC probes. GFP-channel was acquired simultaneously with RNA-FISH imaging, before DNA-FISH. Scale bar: 10 µm. Bottom right panel: GFP nuclear intensity histogram. **F)** Mean GFP intensity in circular areas centered on Subtel DNA-FISH signals (radius = 1.08 µm) for alleles classified as active or inactive regarding *TERT* transcription. Allele transcription status was determined according to the presence or absence of an RNA-FISH signal at a distance below 2.5 µm. Data obtained from RNA/DNA-FISH images from HeLa cells stably expressing dCas9-3XGFP-CRY2, transfected with none or sgSubtel and sgTERT sgRNAs, and illuminated with blue light for 4 h (1 s pulses every 10 s). Black lines represent the median of 400-1500 alleles analyzed per condition. Differences were not statistically significant.

Journal of Cell Science • Supplementary information

Table S1. List of plasmids.

Plasmid	Source	Reference
pCMV-CRY2olig-mCherry	Addgene #60032	(Taslimi et al., 2014)
pCMV-mCherry-CRY2clust	Addgene #105624	(Park et al., 2017)
pCMV-CRY2high-mCherry	Addgene #104063	(Duan et al., 2017)
pCMV-CRY2hiclu-mCherry	Site-directed mutagenesis	Deposited in Addgene (#249577)
pHAGE-dCas9-3XGFP	Addgene #64107	(Ma et al., 2015)
pHAGE-dCas9-3XGFP-CRY2	High-fidelity assembly cloning	Deposited in Addgene (#249578)
pHAGE-dCas9-3XmCherry	Addgene #64108	(Ma et al., 2015)
pHAGE- dCas9-mCherry-CRY2	PCR/restriction cloning	Deposited in Addgene (#249579)
pHAGE-dCas9-3XmCherry-CRY2	High-fidelity assembly cloning	Deposited in Addgene (#249580)
pHR-mCherry-CRY2wt	Addgene #101221	(Shin et al., 2017)
pHR-mCherry-CRY2olig	Addgene #101222	(Shin et al., 2017)
pEF1a-dCas9-CIBN	Addgene #127664	(Kim et al., 2019)
pHAGE-dCas9-3XGFP-CIBN	PCR/restriction cloning	Deposited in Addgene (#249581)
psPAX2	Addgene #12260	Gift from Didier Trono (unpublished)
pMD2.G	Addgene #12259	Gift from Didier Trono (unpublished)

Journal of Cell Science • Supplementary information

Table S2. List of single-guide RNAs. Ordered as synthetic RNAs from Synthego.

Name	Spacer sequence	# repeats	Location
sgIDR3	UGAUGAGCAGAUGUAGGAGG	34	chr19: 380,831-382,654
sgTCF3	AAGGGGACAGCAGAGCUCAC	35	chr19: 1,627,754-1,628,973
sgTelo	UUAGGGUUAGGGUUAGGGUU	~10^2-10^3	Telomeres
sgSubtel1	CGAUGAGAGGCCGGGUGGUC	32	chr5: 83,314-85,466
sgSubtel2	CGGGAGGCGGAGCUCACA	97	chr5: 258,622-262,350
sgTERT1	GUGUGCUGGGGGCUCACUGG	58	chr5: 1,069,995-1,073,282
sgTERT2	UGUGUCUGUAGAGGAGGAGC	35	chr5: 1,326,332-1,328,969

Table S3. List of BACs for DNA FISH. Ordered labeled and ready to use from Empire Genomics.

Name	Clone	Dye	Location (hg19)
IDR3	RP11-575H1	Cy5-dUTP	chr19: 258,325-423,094
TCF3	RP11-317H11	Fluorescein-dUTP	chr19: 1,380,124-1,544,933
Subtel	RP11-44H14	Fluorescein-dUTP	chr5:93,896-269,773
TERT	RP11-117B23	Cy5-dUTP	chr5:1,207,044-1,369,139

Journal of Cell Science • Supplementary information

Table S4. List of nascent RNA FISH probes for TERT. Ordered as a pool and labeled with Atto565 from LGC Biosearch Tecnhologies.

Probe sequence	Target
ccgaccccggggaggcccac	Intron 1
cacgtgacgatggagacagg	Intron 2
tcgacgtcctgagcgaaaag	Intron 2
gaacctcgtaagtttatgca	Intron 2
aaaccgcgtgtccatcaaaa	Intron 2
attataggtaacctgcaggc	Intron 2
caccccttgaaattgcgaaga	Intron 2
tgtgctggagaacagtctta	Intron 2
ccctcttcatacctaaagat	Intron 2
ggttcctcaacatcaaatcc	Intron 2
ggtgaaatcgggacttcttc	Intron 2
tcttacatgtcttgggagtt	Intron 2
catgacgcttatctgactcg	Intron 2
cgaaggaagctggagcacaa	Intron 2
agtcctgatcagagaactca	Intron 2
ctcaagtgagcaaacgccaa	Intron 2
aaaagataggcttggggacc	Intron 2
aatggaacggagaggtgacc	Intron 2
caagctgggagaggagtatt	Intron 2
agagaaccctttctgggatg	Intron 2
gcagcttgggagaaaacagg	Intron 2
tgcccagaataaggtgacaa	Intron 2
tggacgtcaatccatgtgag	Intron 2
ctaagacccaagagggaagt	Intron 2
gcacttagagggaaaggcat	Intron 2
tcaaaacacagggtgcaggt	Intron 2
gtggctgatgttgagattac	Intron 2
caggtaaagctggggtttac	Intron 2
tgtgcatcataagcagaggt	Intron 2
cactcactggctaaggaacg	Intron 2
caggtttgcgcgatttcaaa	Intron 2

Journal of Cell Science • Supplementary information

SUPPLEMENTARY REFERENCES

Duan, L., Hope, J., Ong, Q., Lou, H. Y., Kim, N., McCarthy, C., Acero, V., Lin, M. Z. & Cui, B. 2017. Understanding CRY2 interactions for optical control of intracellular signaling. *Nat Commun,* 8**,** 547.

Kim, J. H., Rege, M., Valeri, J., Dunagin, M. C., Metzger, A., Titus, K. R., Gilgenast, T. G., Gong, W., Beagan, J. A., Raj, A., et al. 2019. LADL: light-activated dynamic looping for endogenous gene expression control. *Nat Methods,* 16**,** 633-639.

Ma, H., Naseri, A., Reyes-Gutierrez, P., Wolfe, S. A., Zhang, S. & Pederson, T. 2015. Multicolor CRISPR labeling of chromosomal loci in human cells. *Proc Natl Acad Sci U S A,* 112**,** 3002-7.

Park, H., Kim, N. Y., Lee, S., Kim, N., Kim, J. & Heo, W. D. 2017. Optogenetic protein clustering through fluorescent protein tagging and extension of CRY2. *Nat Commun,* 8**,** 30.

Shin, Y., Berry, J., Pannucci, N., Haataja, M. P., Toettcher, J. E. & Brangwynne, C. P. 2017. Spatiotemporal Control of Intracellular Phase Transitions Using Light-Activated optoDroplets. *Cell,* 168**,** 159-171 e14.

Taslimi, A., Vrana, J. D., Chen, D., Borinskaya, S., Mayer, B. J., Kennedy, M. J. & Tucker, C. L. 2014. An optimized optogenetic clustering tool for probing protein interaction and function. *Nat Commun,* 5**,** 4925.

Journal of Cell Science • Supplementary information
